# Point-of-Care Ultrasound in the Diagnosis of an Incarcerated Inguinal Hernia

**DOI:** 10.7759/cureus.16281

**Published:** 2021-07-09

**Authors:** Kristina Jacomino, Sarah E Frasure, Keith S Boniface, Hamid Shokoohi

**Affiliations:** 1 Department of Emergency Medicine, George Washington University School of Medicine and Health Sciences, Washington, DC, USA; 2 Department of Emergency Medicine, Massachusetts General Hospital, Harvard Medical School, Boston MA, USA

**Keywords:** point-of-care ultrasound, inguinal hernia, small bowel obstruction, abdominal pain, scrotal pain

## Abstract

Emergency physicians can use point-of-care ultrasound to diagnose inguinal hernias as well as their potential complications, including small bowel obstruction, incarceration, and even strangulation. We provide an overview of the sonographic appearance of inguinal hernias, as well as the diagnostic criteria of serious complications. In this case report, point-of-care ultrasound findings included a non-reducible inguinal hernia associated with significant bowel dilation in multiple loops without signs of intestinal ischemia or necrosis.

## Introduction

The Emergency Department (ED) assessment of patients with suspected inguinal hernia involves establishing the diagnosis and classifying patients into the category of uncomplicated reducible hernia and those with incarceration and/or strangulation. In cases with an incarcerated hernia, it is imperative to determine if it is associated with strangulation, which leads to bowel necrosis, or if there are other complications, such as a small bowel obstruction. Certainly, a small bowel obstruction secondary to a hernia is one of the most frequent causes of abdominal pain requiring surgical intervention [1]. Patients who suffer a delay in diagnosis and treatment of incarcerated or strangulated hernias have higher rates of postoperative complications, such as wound infections, gastrointestinal bleeding, pseudomembranous colitis, and death [2-3]. Computed tomography (CT) imaging is currently the main imaging modality to diagnose a hernia, with a sensitivity of 90% and a specificity of 97% [4]. Point-of-care ultrasound (PoCUS), however, is another effective imaging modality to assess for an inguinal hernia, and it can also detect possible strangulation or concomitant small bowel obstruction [5]. Ultrasound findings that suggest a small bowel obstruction include dilated loops of bowel with a maximum diameter > 2.5 cm and/or "back-and-forth" peristalsis [6]. Signs that should alert the clinician towards a possible diagnosis of strangulated bowel include the lack of dopplerable color flow within the incarcerated hernia, echogenic fat stranding, and a thickened bowel wall. Emergency physicians should be aware of this modality as it can be performed rapidly at the bedside, can accelerate surgical consultation, and thus ensure expedited management. We present the case with a patient who was diagnosed with an incarcerated inguinal hernia and associated small bowel obstruction by using PoCUS. We also describe the proper sonographic protocol and potential pitfalls for the clinician to be aware of.

## Case presentation

A 43-year-old male, with a history of a prior indirect inguinal hernia that did not require surgical intervention, presented to the ED with acute right scrotal swelling that had commenced three hours prior to his ED arrival. The patient, a construction worker, had been lifting heavy objects at work and noted the sudden onset of lower abdominal pain, associated with the swelling of his right hemi-scrotum. He was nauseous but denied vomiting, back pain, dysuria, hematuria, fever/chills, or diarrhea. He had no chronic medical conditions and took no medications.

On physical examination, the patient appeared in significant discomfort. Although there was no abdominal tenderness to palpation, the right hemi-scrotum was markedly swollen and exquisitely tender to palpation. His physical examination was otherwise normal including vital signs that were within normal limits. His laboratory results revealed a white blood cell count of 12.9 x 10^9^/L (normal: 4.5 to 11 x 10^9^/L) and a lactic acid of 2.16 mmol/L (normal: < 2 mmol/L).

**Figure 1:**
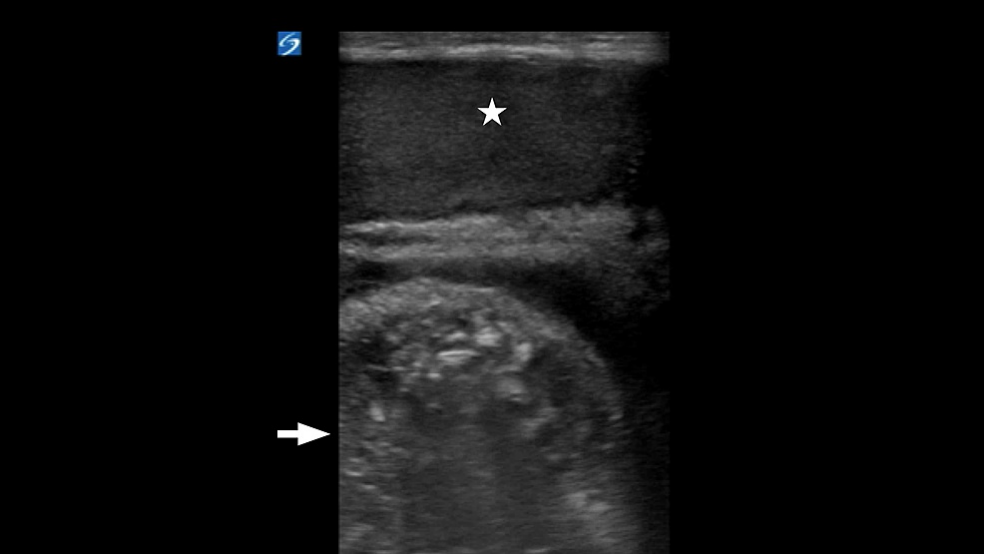
Normal-appearing testicle noted at the top of the screen (star). One loop of bowel was noted posterior to the testicle (arrow).

**Figure 2:**
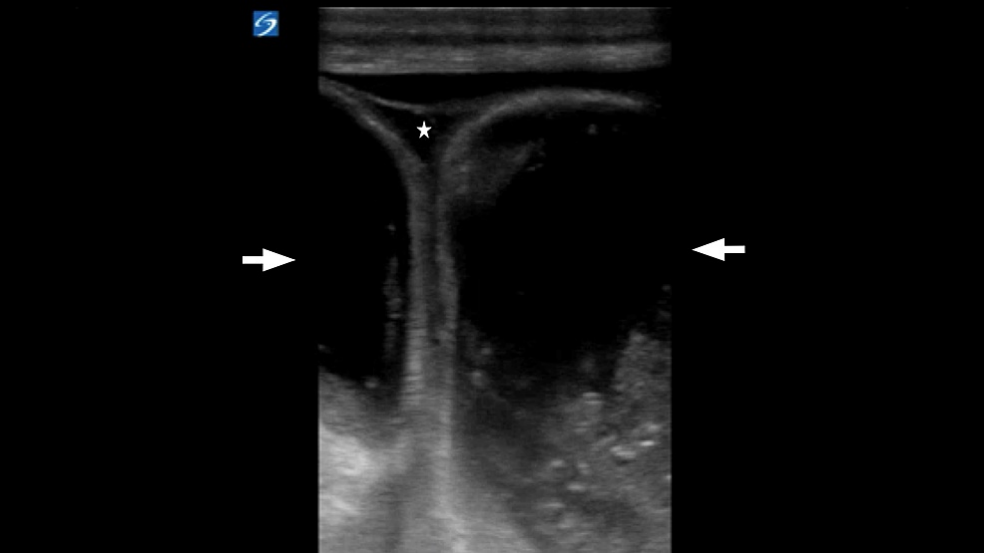
Fluid-filled loops of bowel (arrow) with interloop free fluid (star), concerning for a small bowel obstruction (SBO), are noted in the hernia sac.

**Figure 3:**
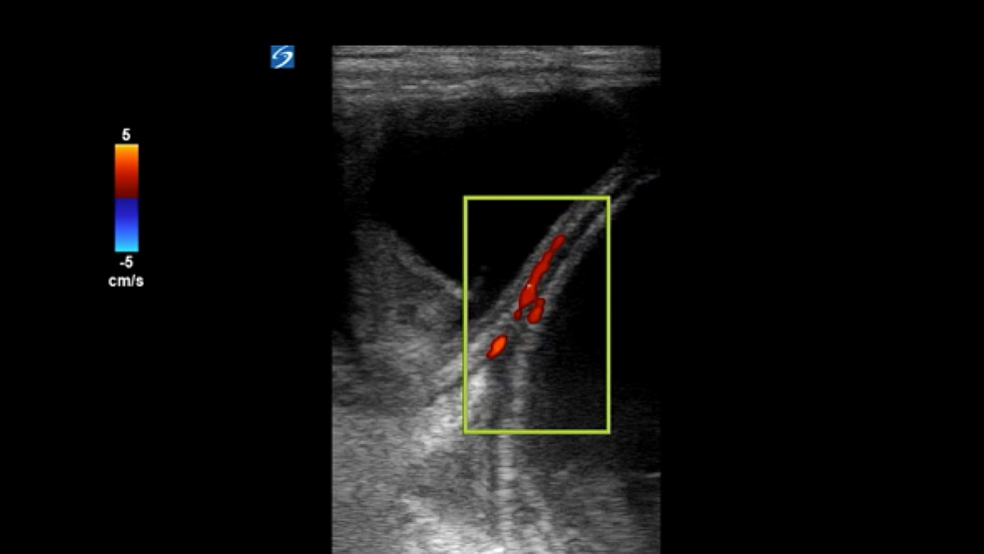
Preserved vascular flow in the bowel wall is identified via Doppler imaging.

The emergency physician performed a PoCUS of the scrotum to further assess the etiology of the patient’s severe pain. Using the linear transducer (SonoSite L25xp; 13-6) (FUJIFILM Sonosite, Inc., Bothell, WA), the clinician scanned the right testicle in the longitudinal and transverse position. The testicle appeared normal, but dilated loops of the bowel were noted in the scrotum (Figures 1-2). Although no peristalsis was appreciated within the dilated bowel loops, the physician identified pulsatile blood flow within the bowel wall by using Doppler imaging, making bowel strangulation/infarction unlikely (Figure 3). Additionally, there was no evidence of bowel wall thickening or free air in the intestinal wall. An attempt at immediate reduction of the inguinal hernia was unsuccessful. The general surgery service was emergently consulted. They were also unable to reduce the hernia at the bedside. The patient subsequently underwent CT imaging which identified a large right inguinal hernia with dilated loops of bowel, as well as a secondary small bowel obstruction. No pneumatosis or free air was visualized, and there was no evidence of strangulation or intestinal necrosis. The patient proceeded to the operating room where the dilated loops of viable small bowel were removed from the scrotum and a mesh was placed to repair the abdominal wall defect. The patient was discharged from the hospital a few days later in good condition.

## Discussion

When using US to detect inguinal hernias, the high-frequency linear transducer is normally used, given the superficial nature of the structures that the clinician needs to visualize. Both the soft tissue of the abdominal wall and the native scrotal structures are examined and evaluated. Sometimes even small indirect inguinal hernias can be seen lateral and superficial to the inferior epigastric artery and descending downwards towards the scrotal sac [5]. Sonographic signs of hernia incarceration in the groin US include free fluid in the hernia sac or within the hernia bowel loop, bowel wall thickening, and dilated bowel loops [7]. In Figure 2, fluid-filled loops of the bowel and inter-loop fluid are seen in the hernia sac, concerning a small bowel obstruction. Normally, the testes and epididymis will remain unchanged, with the aforementioned findings around them, as noted in Figure 1. Demonstrating normal vascular flow via Doppler imaging within the loops of the bowel of an incarcerated inguinal hernia may predict bowel viability [8]. Preserved vascular flow is identified in this manner in Figure 3. In our case, the physician performed a PoCUS to examine the right testicle and identified multiple dilated loops of bowel in the scrotum, making the diagnosis of an inguinal hernia. Although there was no bowel wall peristalsis, the physician was able to identify blood flow in the bowel wall with no significant bowel wall thickening, making a diagnosis of strangulated bowel less likely. However, the clinician identified multiple dilated loops of bowel > 2.5 cm with a lack of peristalsis and small interloop free fluid, compatible with the diagnosis of small bowel obstruction (SBO). When immediate hernia reduction at the bedside was unsuccessful, the clinician obtained a surgical consult and expedited CT imaging. Although CT imaging remains the imaging of choice in the determination of a hernia and its various complications, PoCUS is an important adjunct in a clinician’s armamentarium when exploring potential causes of acute abdominal pain and can be particularly useful in the assessment of ED patients with suspected inguinal hernia.

CT and MRI are the imaging modalities of choice when evaluating hernias. In the ED, CT is utilized much more frequently than MRI, however, given its relative speed and availability. Yet, PoCUS is also often readily available, less expensive, uses no ionizing radiation, and allows for dynamic assessment. Furthermore, in the developing world, US is often the only imaging option to evaluate patients with abdominal pain [9]. In one study that compared CT/MRI as the gold standard, the overall sensitivity, specificity, and accuracy of US for the diagnosis of a groin hernia were all found to be 96% [10]. The authors also analyzed US reliability using the postoperative diagnosis as the gold standard, which demonstrated a sensitivity, specificity, and accuracy of US for diagnosing the presence of a groin hernia were 97%, 67%, and 96%. Another study suggested that in detecting groin hernias specifically, US had a sensitivity and specificity of 92.7% and 81.5%, as compared to 94.5% and 96.3% for MRI [11]. However, using PoCUS in the ED to diagnose incarcerated inguinal hernias remains underutilized and under-evaluated. US can also be employed to diagnose and assist in the reduction of an incarcerated inguinal hernia [12-13]. Yet, no studies have examined the sensitivity and specificity of US in the diagnosis of incarcerated inguinal hernias presenting to the ED. Finally, US is also an effective tool to examine the presence of an SBO in patients with a hernia. Gottlieb et al. demonstrated that US use in the ED had a sensitivity of 93% and a specificity of 96% (with a +LR (likelihood ratio) of 21 and a −LR of 0.08) in the diagnosis of an SBO [6].

## Conclusions

In this case, an emergency physician performed a scrotal ultrasound which identified dilated loops of bowel in the scrotal sac with evidence of preserved bowel wall blood flow, suggesting an incarcerated hernia that was not yet strangulated. As a result, the emergency physician was able to quickly mobilize the surgical consult service and expedite the patient’s care. PoCUS is a truly valuable tool in the ED for the rapid assessment and diagnosis of patients who present with abdominal and/or scrotal pain.
